# Estimating mouthing exposure to chemicals in children’s products

**DOI:** 10.1038/s41370-021-00354-0

**Published:** 2021-06-29

**Authors:** Nicolò Aurisano, Peter Fantke, Lei Huang, Olivier Jolliet

**Affiliations:** 1 Quantitative Sustainability Assessment, Department of Technology, Management and Economics, Technical University of Denmark, Produktionstorvet 424, 2800 Kgs. Lyngby, Denmark; 2 Department of Environmental Health Sciences, School of Public Health, University of Michigan, 1415 Washington Heights, Ann Arbor, MI 48109 USA

## Abstract

**Background:**

Existing models for estimating children’s exposure to chemicals through mouthing currently depends on the availability of chemical- and material-specific experimental migration rates, only covering a few dozen chemicals.

**Objective:**

This study objective is hence to develop a mouthing exposure model to predict migration into saliva, mouthing exposure, and related health risk from a wide range of chemical-material combinations in children’s products.

**Methods:**

We collected experimental data on chemical migration from different products into saliva for multiple substance groups and materials, identifying chemical concentration and diffusion coefficient as main properties of influence. To predict migration rates into saliva, we adapted a previously developed migration model for chemicals in food packaging materials. We also developed a regression model based on identified chemical and material properties.

**Results:**

Our migration predictions correlate well with experimental data (*R*^2^ = 0.85) and vary widely from 8 × 10^−7^ to 32.7 µg/10 cm^2^/min, with plasticizers in PVC showing the highest values. Related mouthing exposure doses vary across chemicals and materials from a median of 0.005 to 253 µg/kg_BW_/d. Finally, we combined exposure estimates with toxicity information to yield hazard quotients and identify chemicals of concern for average and upper bound mouthing behavior scenarios.

**Significance:**

The proposed model can be applied for predicting migration rates for hundreds of chemical-material combinations to support high-throughput screening.

## Introduction

During their growth, children mouth a variety of products, such as toys, teethers, and pacifiers, which all contain various chemical substances. In fact, during the manufacturing process of children’s products, a wide range of chemical additives is used to obtain or optimize specific properties (e.g., plasticizers, flame retardants, antimicrobials) [1–4]. For this reason, there are public concerns about the possibility of children’s products containing toxic chemical substances and exposing children to potentially harmful chemicals [5, 6]. These include, but are not limited to, phthalate plasticizers [7–9], brominated flame retardants [10–12], fragrance allergens [13], as well as non-intentionally added substances [14], which may all pose health risks, especially to children [15, 16]. Since many of these additives are not covalently bound to the polymer chains, during mouthing they might migrate from the products into saliva [14, 17]. Typical examples are plasticizers, such as phthalates and their alternatives, in polyvinyl chloride (PVC) products [18].

Children have a characteristic behavior (e.g., mouthing of articles) and are considered particularly sensitive to chemicals exposure due to their high surface area to body weight ratio, fast metabolic rate as well as the fast growth of organs and tissues [19, 20], with potentially higher responses associated with the development of key developmental and cognitive functions. Hence, when assessing the exposure of this population sub-group, it is crucial to include children-specific exposure pathways, such as mouthing. Nevertheless, across different frameworks and tools, such as chemical and product safety assessment, life cycle impact assessment (LCIA), chemical alternatives assessment (CAA), and prioritization for further risk evaluation, mouthing as an exposure pathway is often poorly quantified, or completely neglected and needs to be better considered [21]. Throughout the present study, the term “mouthing exposure” thereby refers—unless state otherwise—to direct mouthing of children’s products (object-to-mouth). In addition, the focus of this study is on children’s products, thus other product types (e.g., furniture, flooring materials) are not directly addressed.

There are several experimentally based approaches available for quantifying mouthing exposure for children [22]. These approaches are usually quite consistent, straightforward, but require at least four parameters: measured chemical migration rate from the specific product into saliva, mouthing surface area, mouthing time and body weight of the child. As highlighted by an Organisation for Economic Co-operation and Development’s recently published study [22], these approaches are fully dependent on the limited availability of experimental migration rates that are not only chemical-specific but also dependent on the material matrix. Thus, in case of lack of migration rates for a particular chemical-material combination, they become unserviceable.

Measured chemical migration rates from products into saliva have generally covered only a handful of chemicals (mostly phthalates and brominated flame retardants as chemical groups) [10, 23]. Moreover, for these chemicals, only combinations with few types of materials are studied (e.g., PVC and silicone products). Nevertheless, the type of material might have a significant influence on the chemical migration rate [19]. Considering the wide range of chemical-material combinations of children’s products currently being marketed, other potentially relevant chemical groups and material types, which may all pose health risks to children, remain completely unaddressed. Since conducting migration experiments for each different chemical-material combination is costly and time-consuming, mathematical estimation methods are needed, generalizing the experimental knowledge and data acquired on particular chemicals and products, to estimate the migration rates for thousands of marketed chemical-product combinations.

Thus the aim of this study is to develop a (high-throughput suited) mouthing exposure model to estimate migration from article into saliva and subsequent mouthing exposure for chemicals in children’s products made of different materials. To achieve this aim, we focus on four specific objectives:

1. to collect experimental data on chemical migration rates into saliva for different chemical groups and materials;
2. to identify main properties influencing chemical migration rates into saliva;
3. to develop and evaluate a predictive high-throughput model for migration into saliva and related mouthing exposure valid across a large range of material-chemical combinations, and
4. to derive a set of exposure and related risk estimates for mouthing of children’s products for average and upper bound mouthing behavior scenarios and compare them to other exposure pathways for chemicals with available experimental data.

The proposed mouthing exposure model will be suitable for integration into various exposure and impact assessment frameworks, including the multi-pathway near/far-field Product Intake Fraction (PiF) framework, for application in high-throughput risk screening, LCIA, and CAA for chemical substitution and risk prioritization [24–26].

## Methods

### Review of experimental migration data into saliva

We first gathered available peer-reviewed studies reporting chemical migration rates from different products into saliva. We included only studies providing information on both initial chemical concentration in the product and on the amount of chemical migrated into saliva, requisites essential for deriving a migration rate. The collection of migration rates from a study often required standardization of the reported results due to the lack of standard test methods, consistent units, and different tested products. We standardized all chemical migration rates into a consistent unit of μg/10cm^2^/min, the 10 cm^2^ representing the typical mouthing area of children [22]. When migration rates were not directly reported, we derived them based on the information reported on the experimental settings (contact time, sample size) and the reported results (final chemical concentration in saliva, fraction migrated), see the Supplementary Information (SI), section S-1 for more details.

We harmonized and structured all the information collected in a dataset providing for each chemical-material combination the specific migration rate, the initial chemical concentration, the type of material tested and other testing information, such as contact duration and dimensions of the sample. The variety of materials tested in the considered studies included wood, PVC, polypropylene (PP), ethylene-vinyl acetate (EVA), and silicone. An overview of the considered studies is provided in the SI (Table S1).

### Assessing properties potentially influencing migration rates

Various factors might influence the migration rates of chemicals from children’s products into saliva. Migration rates depend on both the specific physicochemical properties of the chemical and the characteristics of the material from which it is migrating. First, numerous studies highlighted a direct correlation between the chemical concentration in the product and the migration rate, the higher the concentration within a given material, the higher the migration rate [23, 27]. However, non-linear saturation phenomena have also been observed for high formulation contents, for example, for the release of phthalates from PVC [18].

Other studies have shown an inverse correlation between migration rates and molecular weight (*MW*), octanol-water partitioning coefficient (*K*_ow_), and water solubility of the chemical [18, 23, 28]. Other factors influencing the migration rates include the type of material containing the chemical (e.g., different types of polymers), the characteristics of the children’s product (e.g., surface roughness, coating type, or thickness) and the specific chemical bonds [19, 22, 27, 28]. Therefore, migration rates of the same chemical at equal concentrations in two different materials are expected to differ. In order to identify the properties potentially influencing chemical migration rates into saliva, we will conduct an explanatory analysis of different chemical properties and experimentally measured migration rates. In addition to the chemical-specific parameters, we plan to evaluate how combined material-chemical properties, such as diffusion coefficient and material-water or -saliva partition coefficient might be able to explain the combined influence of material and chemical properties, as observed for the release of chemicals from building products or toys [21, 29, 30]. The evaluation will be conducted using the previously described experimental migration data collected, the chemical and material-specific properties data and by applying the following presented models.

### A model for predicting migration into saliva

The chemical migration process from solid products to saliva during mouthing is similar to the migration process from food contact materials to food, since saliva can be considered a special type of liquid food. We therefore propose to adapt and test as a mouthing model, the high-throughput suited migration model developed by Ernstoff et al. [31] for chemicals in food packaging, which accounts for two key input parameters: the chemical diffusion coefficient in the packaging material (or other solid product) *D*_p_ (cm^2^/s), and the packaging-food partition coefficient *K*_pf_ (−). These parameters can be predicted for multiple material-chemical combinations using regression QSPRs as functions of the material (Table S2) and *MW* for *D*_p_ [32], and functions of material (Table S3), *K*_ow_ and the ethanol equivalency (EtOH-eq) of the food (here saliva) for the material-saliva partition coefficient *K*_ms_ (−) [30]. This model is therefore applied in the present study to predict the migration from children’s products to saliva, as a combination of a short-term diffusion-dominated model and a longer-term two exponentials saturation model. The migration process is dominated by diffusion inside the product at the start of the migration process, while it is dominated by partitioning between the product and saliva in the longer-term, as reflected in the two exponentials saturation model. As a starting point, we estimate the time of deviation from the simple diffusion. If the mouthing duration is shorter than this deviation time, the short-term diffusion model is applied; otherwise, the longer-term two exponentials saturation model considering both diffusion and partitioning is applied. Detailed equations for the model and parameters estimation are provided in Table 1 and in the SI, section S-2.

**Table 1:** Adapted migration model and required parameters developed to predict chemical migration from children’s products to saliva [30].

Model	$$f_{mgr} \,=\, \left\{ {\begin{array}{*{20}{c}} {f_{ts},\;if\; t \,\le\, t_{dev}} \\ {f_{tdev} \,+\, \left( {\frac{\alpha }{{1 \,+\, \alpha }} \,-\, f_{tdev}} \right) \cdot \left( {A \cdot \left( {1 \,-\, e^{ - B \cdot \beta \cdot \left( {t \,-\, t_{dev}} \right)}} \right) \,+\, \left( {1 \,-\, A} \right) \cdot \left( {1 \,-\, e^{ - C \cdot \beta \cdot \left( {t \,-\, t_{dev}} \right)}} \right)} \right),\;if\; t \,> \, t_{dev}} \end{array}} \right.$$
Parameters	$$t_{dev}= \frac{d_{p}^{2}}{D_{p}}\cdot \left(\frac{0.3552}{1+85.88\cdot e^{{-3.506\cdot {\mathrm{log}}(\alpha)}}}\right),\; if\; \alpha\, > \, 0.2;\; t_{dev}=\frac{d_{p}^{2}}{D_{p}}\cdot 0.0085 \cdot e^{{4.458\cdot {\mathrm{log}}(\alpha)}},\; if\; \alpha \leq 0.2$$$$f_{ts}= \frac{2}{{d_{p}}}\cdot \left(\frac{D_{p}\cdot t}{\pi}\right)^{1/2};\; f_{tdev}=\frac{2}{{d_{p}}}\cdot \left(\frac{D_{p}\cdot t_{dev}}{\pi}\right)^{1/2};\, \alpha = \frac{1}{K_{pf}}\cdot \frac{V_{f}}{V_{p}};\, \beta = \frac{1}{d_{p}}\cdot \sqrt{ \frac{D_{p}}{\pi \cdot t_{dev}}\cdot \left(\frac{\alpha}{1+\alpha}-f_{tdev}\right)}$$$$A \,=\, 0.7\; \textrm{for}\; x_1 \,=\, 10^{0.12 \cdot \log \left( \alpha \right) \,+\, \log \left( {0.8} \right)} \,<\, 0.7;\; 1\; \textrm{for}\; x_1 \,> \, 1;\; x_1\; \textrm{elsewhere}$$$$B \,=\, 0.3\; \textrm{for}\; x_2 \,=\, 10^{0.22 \cdot \log \left( \alpha \right) \,+\, \log \left( {0.5} \right)} \,<\, 0.3;\; 0.9\; \textrm{for}\; x_2 \,> \, 0.9;\; x_2\; \textrm{elsewhere}$$$$C \,=\, 0.004\; \textrm{for}\; x_3 \,=\, 10^{0.7 \cdot \log \left( \alpha \right) \,+\, \log \left( {0.08} \right)} \,<\, 0.3;\; 1\; \textrm{for}\; x_3 \,> \, 1;\; x_3\; \textrm{elsewhere}$$

*t* = the mouthing duration (s), *t*_*dev*_ = the time of deviation from the simple diffusion model (s), *f*_*mgr*_ = the fraction of the product of the chemical originally in the children’s product that is migrated to the saliva after a certain duration (dimensionless); *d*_*p*_ = the thickness of the product (cm); *D*_*p*_ = the chemical’s diffusion coefficient inside the product (cm^2^/s); *k*_*pf*_ = the chemical’s product-food partition coefficient (dimensionless); *V*_*f*_ = the volume of food (here saliva) (cm^3^); *V*_*p*_ = the volume of product (cm^3^).

The developed exposure model focuses on estimating migration into saliva as the intake. It does not aim at modeling the uptake across mouth/GI barriers, but indirectly considers the background influence of the presence of mouth and saliva flux into GI tract on the migration to saliva. When estimating the *K*_ms_ as a function of the EtOH-eq of the saliva, some adaptations might be required to account for the specific characteristic of mouthing, for which saliva is in permanent contact with the rest of the mouth. Due to the close contact between the saliva and the skin and muscles inside the mouth, whose volume is much larger than that of saliva, rapid transfer from chemicals in saliva to skin/muscle is expected, thus an EtOH-eq value of meat might be more appropriate. We will therefore consider two possible values of EtOH-eq: 20% which was suggested as proxy for EtOH-eq of saliva [33], and 50% which is the EtOH-eq of pork [34]. In addition, when comparing the predicted results against experimental values, by considering two different EtOH-eq values we might be able to reflect potential differences in experimental settings (e.g., in vivo against in vitro studies with or without mechanical agitation).

The output of the model is the fraction of the chemical originally in the children’s product that is migrated to the saliva after a certain duration *f*_mgr_ (dimensionless), converted to a migration rate by:

1 $$R_{{\mathrm{mgr}}} \,=\, \frac{{f_{{\mathrm{mgr}}} \,\times\, m_0}}{{A_{{\mathrm{contact}}} \,\times\, t}}$$

Where *R*_mgr_ is the migration rate (µg/10cm^2^/min), *m*_0_ is the initial chemical mass in the product (µg), *A*_contact_ is the mouthing contact area (cm^2^), *t* is the mouthing duration (min).

Alternatively to the predictive mechanistic model, we propose to test multiple linear regression models with forward selection, after identifying through an explanatory analysis the most relevant properties influencing chemical migration to consider as independent variables:

2 $$R_{{\mathrm{mgr}}} \,=\, \beta _0 \,+\, \beta _1x_1 \,+\, \beta _2x_2 \,+\, \ldots \,+\, \beta _ix_i \,+\, {\it{\epsilon }}$$

Where *β*_0_ is the intercept, *β*_*i*_ is the slope for each independent variable and *x*_*i*_ is a chemical and/or material-specific property influencing the chemical migration rate, such as the initial chemical concentration, *MW*, *K*_ow_, or *D*_p_ of the chemical in the considered material. The performances and results of the two models are then analyzed and recommendations provided.

### Quantifying mouthing exposure and related health risk

To quantify mouthing exposure and related risk, we first defined mouthing exposure scenarios. Since the daily mouthing duration is dependent on both the age group and the mouthed object, we considered two distinct age groups of 3 to <6 months and 2 to <3 years, and two distinct types of children’s products represented by a pacifier for products that are meant to be mouthed long-term and a doll for products that are occasionally mouthed. Before performing the exposure assessment, we associated each observation and product-chemical combination of the dataset with either the pacifier or the doll based on the type of product tested during the experiments. Since mouthing duration might vary substantially across children, we also differentiated between an average and a upper bound mouthing behavior corresponding to the 99^th^ %-ile of child mouthing duration [35]. Table 2 summarizes the different mouthing scenarios for quantifying children chemical exposure, while the characteristics of the two children’s products considered are given in the SI (Table S4).

**Table 2:** Mouthing exposure scenarios for the two age groups and children products considered [34].

Age group	Body weight (kg)	Average Mouthing duration (min/h)		99^th^ %-ile Mouthing duration (min/h)	
		Pacifier	Doll	Pacifier	Doll
3 to <6 months	7.4	3.4	0.5	37.3	2.5
2 to <3 years	13.8	1.8	0.4	46.3	2.9

The predicted daily exposure to chemicals through mouthing children’s products (*E*_mouthing_ in μg/kg_BW_/d) is estimated using Eq. 3:

3 $$E_{{\mathrm{mouthing}},{\mathrm{pred}}} \,=\, \frac{{{\Delta} t_{{\mathrm{mouthing}}} \,\times\, A_{{\mathrm{contact}}}\,\times\, R_{{\mathrm{mgr}},{\mathrm{pred}}}}}{{BW}}$$

where, *t*_mouthing_ is the mouthing duration per day (min/d), *A*_contact_ is the mouthing contact area (cm^2^), *R*_mgr,pred_ is the predicted chemical migration rate into saliva (µg/10 cm^2^/min), and *BW* is the body weight of the child (kg).

This predicted exposure dose based on the defined exposure scenarios can first be compared to the experimentally derived exposure dose from papers, calculated from the experimental migration rates *R*_mgr,exp_.

To quantify children’s risk, we then combined exposure estimates with toxicity values for non-cancer effects. *E*_mouthing,pred_ estimates were divided by the reported reference doses (*RfD*) for oral exposure (μg/kg_BW_/d) to determine potential health risks from mouthing children’s products in terms of hazard quotient (*HQ*):

4 $$HQ \,=\, \frac{{E_{{\mathrm{mouthing}},{\mathrm{pred}}}}}{{RfD}}$$

Where available, *RfDs* were obtained from experimental data [36–39], or otherwise predicted using quantitative structure-activity relationship (QSAR) models [40].

## Results

### Measured chemical migration rates into saliva

**Fig. 1:**
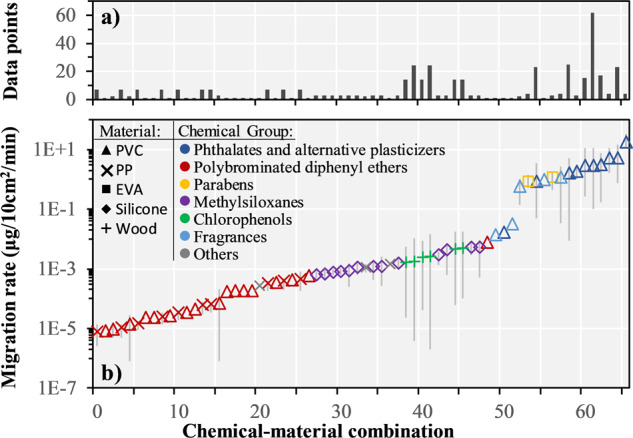
Overview of the built harmonized dataset of collected chemical migration rates into saliva. **a** Number of data points available and **b** migration rates (μg/10cm^2^/min) of the collected chemical-material combinations, ranked by increasing average migration rate (66 chemical-material combinations from 18 studies covering 60 chemicals and 5 materials). Vertical gray error bars indicate the range of migration rates available in the built dataset for each chemical-material combination. EVA: poly(ethylene-co-vinyl acetate), PP: polypropylene, PVC: polyvinyl chloride.

Based on the systematic literature review, we built a dataset of *n* = 437 experimental chemical migration rates into saliva with *n* = 66 unique chemical-material combinations covering *n* = 60 different chemicals in *n* = 5 different materials. Diffusion inside the article and partitioning between the article surface and the saliva depends not only on the chemical but also on the type of material [30, 32]. For this reason, we separately considered migration rates for the same chemical but tested in different materials. Fig. 1 presents an overview of the chemical-material combinations, their average reported chemical migration rates and corresponding ranges across available data points.

The *n* = 437 experimental migration rates collected from the literature span over seven orders of magnitude across chemicals and materials from 1.7 × 10^−6^ μg/10 cm^2^/min for the brominated flame retardant 2,2′,3,4,4′,5′,6-heptabromodiphenyl ether (BDE-183, CAS numbers for all mentioned chemicals are given in the SI, Table S5) in PP to 32.7 μg/10 cm^2^/min for the phthalate plasticizer dibutyl phthalate (DBP) in PVC. High variability was also observed for measured migration rates for the same chemical but in different materials. For example, for the brominated flame retardant decabromodiphenyl ether (DBDE), the reported migration rate in PVC was 8.33 × 10^−6^ μg/10 cm^2^/min, while in PP it ranged from 1.8 × 10^−4^ to 8 × 10^−4^ μg/10 cm^2^/min. Even for the same chemical-material combination, there is sometimes a wide variability in the collected migration rates. For example, for the plasticizer diisononyl phthalate (DINP) in PVC migration rates range from 0.5 to 11.1 μg/10 cm^2^/min across *n* = 62 data points. For this specific chemical-material combination (DINP in PVC), a high number of data points is available in the literature, since it was frequently investigated in the past two decades. The built harmonized dataset with all collected migration rates is provided in the SI (Table S5).

### Main properties influencing migration

#### Initial chemical concentration

**Fig. 2:**
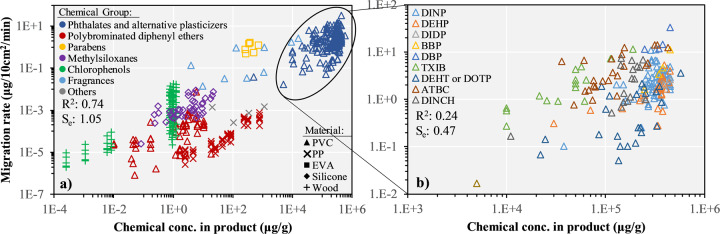
Migration rates as function of initial chemical concentration in the product. **a** differentiating between material and chemical group (*n* = 437) and **b** differentiating between single substances for the phthalates and alternative plasticizers chemical group. EVA: poly(ethylene-co-vinyl acetate), PP: polypropylene, PVC: polyvinyl chloride.

The first parameter of interest that we tested and that is used as the starting point of our analysis is the initial chemical concentration in the children’s product. Fig. 2a presents for the *n* = 437 data points collected the experimental migration rates as function of the initial chemical concentration, differentiating between product materials and chemical groups. As a general trend, the initial chemical concentration and the measured migration rate are positively correlated across materials and chemical groups with a coefficient of determination (*R*^2^) of the log-transformed values of *R*^2^ = 0.74. Similar trends are also observed for each chemical substance as shown in Fig. 2b for selected phthalates and alternative plasticizers. However, even if chemical concentration and migration rate are highly correlated, the residual variations of more than three orders of magnitude need to be further explained for accurately predicting the migration rate of a specific chemical in a specific material. For example, parabens in EVA and polybrominated diphenyl ethers in PP have migration rates differing by more than three orders of magnitude for similar initial concentrations (around 1000 μg/g, Fig. 2a). Within the same material and despite similar chemical contents, migration rates of acetyl tributyl citrate and of di(2-ethylhexyl) terephthalate in PVC differ systematically by two orders of magnitude (Fig. 2b).

#### Chemical-material properties of influence

Three sources suggested that migration rate might be also correlated with *MW* or *K*_ow_ [18, 23, 28]. We studied the correlation between *MW* or *K*_ow_ with measured migration rates and with migration rate divided by the initial chemical concentration (normalized) to account for this main factor of influence. Based on the gathered data, *MW* or *K*_ow_ on their own are not able to explain the variation of orders of magnitude in migration rates between chemicals with similar *MW* and *K*_ow_, such as between methylsiloxanes in silicone versus polybrominated diphenyl ethers in PP (see the SI, Fig. S1).

**Fig. 3:**
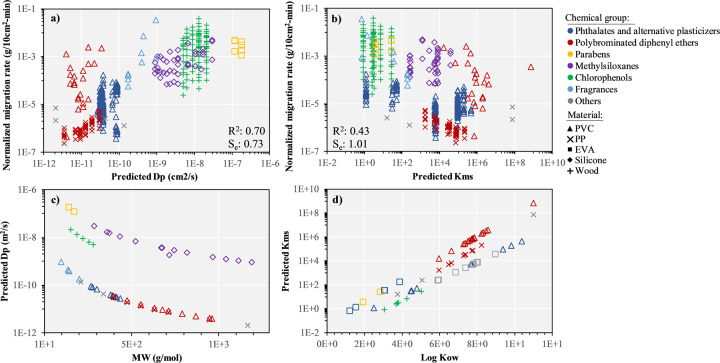
Analysis of chemical-material properties of influence. Normalized measured migration rate as a function of **a** chemical diffusion coefficients within materials *D*_p_ (cm^2^/s) and **b** material-saliva partitioning coefficients *K*_ms_ and **c** *D*_p_ (cm^2^/s) as function of molecular weight (*MW*), and **d** *K*_*ms*_ as function of octanol-water partitioning coefficient *K*_ow_, differentiating between material and chemical group (*n* = 437). EVA: poly(ethylene-co-vinyl acetate), PP: polypropylene, PVC: polyvinyl chloride.

Considering that contact between saliva and children’s product is analogous to the contact between packaging and liquid food, we can hypothesize that the two main parameters driving migration from packaged food [31], namely chemical diffusion coefficients within materials (*D*_p_) and material-food partitioning coefficients (here saliva, *K*_ms_), are also driving migration from children’s product into saliva. To test this hypothesis, we plot the normalized migration rates as a function of *D*_p_ (Fig. 3a) and *K*_ms_ (Fig. 3b). *D*_p_ was estimated as function of *MW* and material type [32], while *K*_ms_ was determined as function of *K*_ow_, material type, and estimated EtOH-eq [30]. For *n* = 2 chemicals we used the high-end limit of the applicability domain of this QSPR [30], i.e., log *K*_ow_ = 11 for estimating *K*_ms_. In addition, to reflect the differences in experimental methods used to derive migration rates, we considered an EtOH-eq = 20% for all in vitro experimental conditions without stimulation (i.e., no physical stimulation of the samples in saliva, contact with glass instead of a mouth), whereas we considered an EtOH-eq = 50% for all experiments performed in vivo or with mechanic agitation of the samples. This reflects that the saliva is not isolated in a tube but in contact with the rest of the mouth, flesh having a higher EtOH-eq than saliva itself.

Figure 3c first shows the dominant influence of materials, with the three orders of magnitude higher diffusion coefficients for chemicals in wood, silicone or EVA, compared to chemicals of similar *MW* in PVC or PP. This is directly reflected in the normalized migration rates: parabens in EVA, methylsiloxanes in silicone and chlorophenols in wood, that are the material-chemical combinations with the higher *D*_p_ values, also show substantially higher normalized migration rates than e.g., phthalates in PVC (Fig. 3a). *K*_ms_ is less influenced by material type and is primarily related to *K*_ow_ (Fig. 3d), with high *K*_ow_ and *K*_ms_ chemicals, such as phthalates or PBDEs showing low normalized migration rates (Fig. 3b). Generally, we observe that normalized migration rates have a positive correlation with *D*_p_ (Fig. 3a, *R*^2^ = 0.70), and an inverse but less significant correlation with *K*_ms_ (*R*^2^ = 0.43). At the same time, we observe also some outliers, such as brominated flame retardants in PVC for *D*_p_ (Fig. 3a).

Figure S1 in the SI presents the normalized migration rate as function of *MW* with substantially lower correlations (*R*^2^ = 0.16, *S*_e_ = 1.22) than for the diffusion coefficient, emphasizing that it is crucial to account for the combined influence of chemical and material properties on diffusion to adequately predict migration. As expected, material plays a lesser role for *K*_ms_ and the correlation between normalized migration rate is primarily related to chemical properties and correlation with *K*_ow_ (*R*^2^ = 0.36) is slightly lower than with *K*_ms_ (*R*^2^ = 0.43).

This analysis of the chemical-material properties of influence suggests that *D*_p_ and *K*_ms_ (or underlying data) are the main parameters to account for, when predicting chemical migration rates to saliva. Building on this result, we propose to test two models; first, we will adapt a food packaging model to saliva migration to obtain a fully predictive model (mechanistic material-saliva migration model). Alternatively, we will conduct a regression model with forward selection of the most important variables among chemical concentration, *D*_p_, *K*_ms_, *K*_ow_, and *MW* (regression-based model).

### Predicted migration rates into saliva

#### Mechanistic material-saliva migration model

The common factors of influence and the analogy between migration in saliva and migration from food packaging suggest that food packaging models could be adapted to estimate chemical migration into saliva, as a predictive model without any parameter fitting. We therefore tested the food packaging model developed by Ernstoff et al. [31] to predict migration rates to saliva as a function of the initial chemical concentration in the mouthed product, *D*_p_, and *K*_ms_ as key input parameters.

**Fig. 4:**
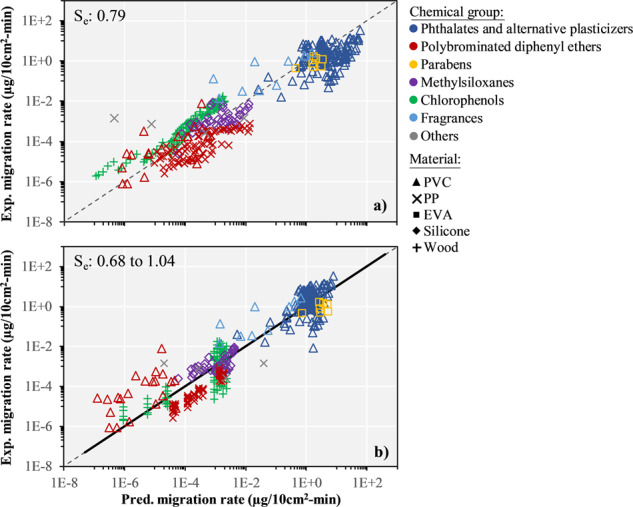
Comparison of the experimental migration rates with the predicted migration rates. **a** presents the predicted migration rates of the mechanistic material-saliva migration model, and **b** the predicted migration rates of the  regression-based model, differentiating between both chemical groups and materials (*n* = 437). The dashed black line represents the 1:1 line, while the solid black line in **b** represents the best fit that also correspond to the 1:1 line. Standard error (S_e_) are evaluated on the log-scale for the 1:1 line, and reported as range in **b** based on the results of the three types of cross-validation. EVA: poly(ethylene-co-vinyl acetate), PP: polypropylene, PVC: polyvinyl chloride.

Figure 4a first compares the predicted and experimental migration rates for all data points in our dataset. The model fits well the experimental results, with a *R*^2^ for the log-transformed values of 0.85 and a standard error (*S*_e_) of 0.79 evaluated for the 1:1 line (see the SI, Table S6 and Fig. S2 for more details on statistics), despite the widely diversified range of experimental studies applying different methods used to test the model. While being fully predictive, without fitting any parameter, this model represents substantial improvements, both in terms of higher *R*^2^ and lower *S*_e_ compared to e.g., the simple correlation with initial chemical concentration (*R*^2^ = 0.74, *S*_e_ = 1.05).

#### Regression-based model

As an alternative to the adapted food packaging model (mechanistic model), the multiple linear regression model using forward parameter selection includes the initial chemical concentration, *D*_p_ and *K*_ow_ as independent variables and is given by:

5 $${\mathrm{log}}_{10}R_{{\mathrm{mgr}}} \,=\, 3.23 \,+\, 0.73\log _{10}D_{\mathrm{p}} \,+\, 0.92\log _{10}C_0 \,-\, 0.06\log _{10}K_{{\mathrm{ow}}}$$

Each of the regression coefficient is highly significant with *p* values lower than 0.0008 (see Table S7 for the statistics of the multiple linear regression). On the other hand, *MW* and *K*_ms_ did not enter the regression with *p* values of 0.79 and 0.17.

The resulting regression coefficient and standard error on the log of *R*^2^ = 0.89 and *S*_e_ = 0.68 (Fig. 4b) cannot be directly compared to the predictive test *R*^2^ and *S*_e_ of the mechanistic material-saliva migration model. To provide comparable standard errors, and to investigate possible influences of artifacts of the dataset on the two models performances, we performed three types of cross-validation (see the SI, Tables S8 and S9), (1) excluding an entire chemical group of the training set (error on predicting migration rates for a new chemical group), (2) excluding entire groups of experiments (to account for experimental bias) or (3) performing a tenfold random cross validation (all experiments and chemical groups are represented in the training and test set). When excluding entire chemical groups (e.g., polybrominated diphenyl ethers) or entire groups of experiments (Table S9), we observed that the predictive performances of the regression-based model (*S*_e_ = 1.04 and 0.96, respectively) are not as good as for the mechanistic material-saliva migration model (*S*_e_ = 0.79).

Overall, the two proposed models work well and give similar results, with the mechanistic model being slightly more conservative for high migration rates observed for phthalates and parabens (see the SI, Fig. S3).

### Mouthing exposure and children risk estimates

We quantified exposure estimates *E*_mouthing_ in μg/kg_BW_/d for each of the *n* = 437 data points in our dataset covering the different chemical-material combinations applying Eq. 3 for the four mouthing scenarios, which consider two children age groups with two daily mouthing durations (Table 2). We allocated each data point to one of the two children’s product considered (i.e., pacifier or doll), modeling all PVC, PP, and wood data as dolls, and silicone and EVA data as pacifiers. All mouthing exposure estimates for the mechanistic material-saliva migration model and the regression-based model are summarized in the SI (Table S5), while results are only presented and discussed in further details in the main text for the mechanistic model.

*E*_mouthing,pred_ widely ranges by eight orders of magnitude across data points and mouthing exposure scenarios (*E*_mouthing,pred_ = 4 × 10^−8^ to 252 μg/kg_BW_/d), with the highest exposures observed for the plasticizers DBP in PVC dolls (*E*_mouthing,pred_ = 21.7–253 μg/kg_BW_/d) and for propylparaben in EVA pacifiers (*E*_mouthing,pred_ = 5.8–224 μg/kg_BW_/d).

**Fig. 5:**
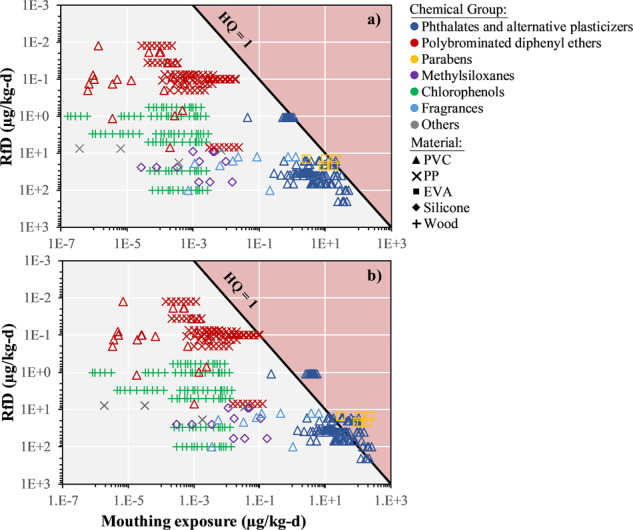
Analysis of mouthing exposure and children risk estimates. Non-cancer reference doses (inverted axis—high to low *RfDs*) as a function of mouthing exposures, for the **a** average and **b** upper bound mouthing exposure duration scenario for 3 to <6 months old children. The black line represent the threshold for hazard quotient of concern (*HQ* = 1). EVA: poly(ethylene-co-vinyl acetate), PP: polypropylene, PVC: polyvinyl chloride.

Figure 5 plots the non-cancer toxicity metric (*RfD*, from high to low *RfDs*) as a function of the daily exposure estimates for the average and upper bound mouthing behavior scenario for a 3 to <6 months old child. The black diagonal line corresponds to an equi-risk level of *HQ* = 1 (*E*_mouthing,pred_ = *RfD*) and delimitates the threshold over which one chemical-material combination becomes of concern, i.e., *HQ* > 1. For the average scenario (Fig. 5a), only three chemical-material combinations out of *n* = 437 data points exceed a *HQ* of 1, namely the two plasticizers 2,2,4-Trimethyl-1,3-pentanediol diisobutyrate (*HQ* = 1.25) and diisononyl cyclohexane-1,2-dicarboxylate (*HQ* = 1.06) in PVC dolls, and propylparaben (*HQ* = 1.39) in EVA pacifiers. Risk is even smaller for the average mouthing scenario with a 2 to <3 years old child (no combinations with *HQ* > 1, see the SI, Fig. S4a). In contrast, risk levels are substantially higher for the upper bound mouthing behavior scenario, with *n* = 70 chemical-material combinations showing a *HQ* of concern for 3 to <6 months old children (1 < *HQ* < 15.2, Fig. 5b) and *n* = 58 for 2 to <3 years old children (1 < *HQ* < 10.1, see the SI, Fig. S4b).

The regression-based model leads to similar results, on average lower by a factor of 3. Only a few phthalates and alternative plasticizers and parabens were found of concern across the four mouthing exposure scenarios (see the SI, Fig. S5). The remaining chemical groups, in the considered materials, showed *HQ* < 1 even in the upper bound mouthing behavior scenario. The detailed risk assessment results are presented in the SI (Table S5).

## Discussion

### Applicability and limitations of the proposed model

The presented high-throughput mechanistic mouthing exposure model, adapted from a previously developed food packaging model, can be applied to a wide range of materials and chemicals present in different children’s products.

The mechanistic material-saliva migration model is able to predict chemical migration rates from different materials into saliva for multiple chemical and material properties, without the need for specific experimental data. Thus, hundreds of chemical-material combinations at different concentrations could be analyzed and compared. In addition, the proposed model is suitable for implementation into the multi-pathway near/far-field PiF framework [24–26], with direct application in different tools, such as high-throughput risk screening, LCIA, and chemical substitution.

Alternatively to the mechanistic material-saliva migration model, we also proposed and tested a regression-based model for estimating migration rates. The regression-based model provides similar performance and is well suited for predicting migration rates into saliva for chemical-material combinations covered in the gathered dataset and used for training the model. However, the regression-based model showed slightly lower predictive power (slightly higher standard error) for new chemical-material combinations, especially when excluding entire chemical groups (e.g., polybrominated diphenyl ethers) during the three types of cross-validation (see the SI, sections S-5 and S-6). Also the regression model coefficient might be sensitive to the underlying quantitative property-property relationship used to predict the input diffusion and partition coefficients. We therefore suggest to apply in priority for new chemical-material combinations (not covered in our dataset) the mechanistic material-saliva migration model since it is fully predictive without any parameter fitting, is proportional to the initial chemical concentration and shows good predictive powers for a large range of chemical-material combinations. While we suggest the application of the regression-based model more in case of chemical-material combinations covered by its training dataset. In addition, we also recommend as a conservative approach the application of an EtOH-eq = 50% in the migration mechanistic model, enabling the correction of in vitro experiment to account via reduced *K*_*ms*_ for the potentially higher transfer from the article not only to saliva but also to the rest of the mouth. The mechanistic material-saliva migration and the regression-based models are provided as a Microsoft Excel workbook (see the SI, section S-11).

The mechanistic material-saliva migration model also comes with limitations. Even though inorganic chemicals, such as heavy metals have been reported in toys, teethers, and feeding teats [41], the model is only able to estimate exposure for organic chemicals, since related migration models for inorganic chemicals are currently lacking [42]. Moreover, the model estimates also depend on the material-saliva partitioning coefficient (*K*_ms_), determined as a function of *K*_ow_ and therefore sensitive to the different *K*_ow_ estimation methods. The model focuses only on leachable compounds from the products to saliva and does include neither ingestion of small pieces (scrapped-off plastic toy material) during the mouthing nor object-hand-mouth contacts that are also relevant pathways that might have an important contribution to the total mouthing exposure [43, 44] and need to be considered separately. Moreover, the model would need specific adaptations (ideally based on experimental data) to cover other applications, such as fabrics due to the potentially different chemical transfer during the circulation of saliva within the textile or as children’s products with surfaces coated with organic films, which might influence specific chemical-material properties, such as *K*_ms_.

### Comparison with other studies and exposure pathways

We first compared our mechanistic results with mouthing exposure estimates present in the literature. Even though there might be differences in specific parameters of the exposure scenarios (e.g., assumed mouthing time or *BW*), we observe that mouthing exposure results are generally in line with results from previous studies. For example, Ashworth et al. [45] estimated for seven phthalates present in plastic toys mouthing daily exposure ranging up to 46 μg/kg_BW_/d for 6 months old children. For the same chemicals, our average estimates range between 0.01 and 50 μg/kg_BW_/d. Babich et al. [23] estimated mouthing exposure for five plasticizers between 0.2 and 9.5 μg/kg_BW_/d, to compare with our estimates for the same chemicals of between 0.02 and 30 μg/kg_BW_/d. Chen et al. [10] calculated mouthing exposure for brominated flame retardants in children’s toys ranging between 5 × 10^−7^ and 5 × 10^−3^ μg/kg_BW_/d, which is very similar to this paper’s estimates between 6.6 × 10^−7^ and 2.7 × 10^−3^ μg/kg_BW_/d across the four exposure scenarios. Finally, for four fragrance allergens, Masuck et al. [13] estimated a mouthing exposure between 1.1 and 22.2 μg/kg_BW_/d, while for the same chemicals our results are slightly lower between 3 × 10^−4^ and 6.3 μg/kg_BW_/d across the four exposure scenarios.

Second, we compared our mouthing exposure estimates with other exposure pathways and products present in the literature. For the flame retardant DBDE a recent study [43] estimated a median hand-to-mouth daily exposure of 2.2 × 10^−4^ ranging up to 4.2 × 10^−3^ μg/kg_BW_/d, estimates close to our average mouthing results. This suggest that both mouthing and hand-to-mouth might be equally relevant pathways. For the plasticizer bis(2-ethylhexyl) phthalate (DEHP) in vinyl flooring, Little et al. [46] estimated exposure for a 3-year-old child equal to 0.013 μg/kg_BW_/d via air inhalation, 75 μg/kg_BW_/d via dust ingestion and 0.12 μg/kg_BW_/d via dermal absorption from air. Compared to our average mouthing estimates (0.01–19.5 μg/kg_BW_/d), these results suggest that the exposure to DEHP from mouthing children’s products and vinyl flooring are within the same orders of magnitude and both important. For the two plasticizers DEHP and DBP another study [47] estimated indoor daily intake via dust ingestion between 0.14 and 5 μg/kg_BW_/d, via air inhalation between 0.03 and 2.1 μg/kg_BW_/d, and via dust dermal uptake between 5 × 10^−4^ and 0.05 μg/kg_BW_/d. For these two phthalates, our average estimates range between 0.01 and 50.6 μg/kg_BW_/d, highlighting again the importance of mouthing as exposure pathways compared to others. This is also supported by the results of another study comparing exposure to PBDEs via inhalation, mouthing, and dermal contact from children’s products and suggesting that mouthing is the predominant pathway for children up to 3 years old [19]. Nevertheless, especially for semi-volatile organic compounds, such as phthalates and alternative plasticizers, when considering all the children’s products present in a household (and not only one single toy), the relevance of this pathway might be limited compared to inhalation, dust ingestion, and dermal gaseous exposure [21]. This is also supported by results from biomonitoring studies suggesting that mouthing might contribute up to one-fourth of young children’s total phthalate exposure [48], highlighting at the same time both the importance of this exposure pathway but also the relevance of all the others.

### Direct comparison of migration rates across experimental studies

Care must be taken when comparing migration rates across chemicals, materials, and studies, because of the lack of a standardized testing method across relevant chemical-material combinations [23]. A standardized testing method on how to conduct saliva migration experiments only exists for phthalates in PVC [18]. Developed by the European Commission Joint Research Centre (JRC), this method assesses the dynamic migration of the chemicals of interest from PVC into artificial saliva via mechanic agitation, using a head over heels device [7, 23, 27]. For other chemicals and materials, the gathered studies implemented different experimental setups. For example, Chen et al. [10] conducted in vivo experiments with adults chewing PVC pieces and collecting saliva samples every 5 min. Other studies implemented an adapted version of the JRC method by cutting the children’s products of interest in small pieces and immerging them in artificial saliva with or without mechanic agitation [49–51]. In our study, we accounted for these differences by standardizing all chemical migration rates into a consistent unit of μg/10 cm^2^/min and by considering an EtOH-eq = 20% for all in vitro experimental conditions without stimulation and an EtOH-eq = 50% for all experiments performed in vivo or with mechanic agitation of the samples. However, other factors, such as temperature, pH and composition of the saliva or saliva simulant (e.g., presences of salts, enzymes), which might also influence the experimental results, were not considered [22, 51]. Nevertheless, the predictions of the mechanistic model are independent on the background data, and in this case the quality of the data is implicitly considered in the overall evaluation. The quality of the considered data has more influence on the regression-based model, thus the need to better standardize experimental set-up and consistently apply it for new migration data. For future experimental studies on chemical migration rates to saliva, we propose the application of a standardized method, such as the JRC method, which will render the comparison and implementation of results across experimental studies more efficient.

### Needs for supplementary data

Regarding the large variability in children’s products materials and their different specific properties, we suggest to extend the coverage of experimentally determined diffusion coefficients and material-water or material-saliva partition coefficients, characterizing in detail both chemical and material properties. These can be used to further develop targeted prediction tools of diffusion and partition coefficient for new materials beyond the 15–19 materials presently covered, as input data for migration modeling. In addition, when new migration rate are determined experimentally, it is crucial to also measure chemical concentrations in children’s products, to be able to take advantage of these data to further develop and evaluate mechanistic models.

Finally, our risk assessment results are highly influenced by the different quality of the gathered toxicity data. Experimental toxicity data were only available for *n* = 23 chemicals, while the remaining chemicals’ reference doses were predicted using QSAR models. Consistently with chemical ingestion via dietary intakes, toxicity experiments provide an aggregated response for both exposure within the mouth and the GI tract. However, as for chemical intakes via food ingestion, there might be a restricted discrepancy in cases the experimental toxicity studies were conducted by gavage which might lead to an underestimation of the chemical toxicity potential in the mouth itself. It is fundamental to provide higher-quality toxicity data and quality-related uncertainty information, also in risk screening-level approaches [52]. Although this type of data selection approaches is emerging for example to estimate freshwater ecotoxicity values [53], systematic methods for data collection and harmonization for human toxicity information are presently still lacking [54].

## Conclusions

By adapting a mechanistic food packaging model, we were able to quantify migration into saliva and subsequent mouthing exposure and risk from chemicals present in children’s products made of different materials. Alternatively to the mechanistic material-saliva model which application is recommended in case of chemical-material combinations not covered by this study, we also proposed and tested a regression-based model more suited for chemical-material combinations covered by its training dataset. The results suggest that mouthing behavior of children has a high influence on mouthing daily exposure and consequently also on the related potential risk. For an average mouthing scenario, we observed potential health risk only for a limited number of chemical-material combinations, while when considering a upper bound mouthing behavior scenario the number of combinations with *HQ* > 1 substantially increased. For this reason, considering only an average mouthing duration when assessing mouthing exposure, as currently done in the majority of the studies available in the literature, might leads to underestimate of the actual risk on children since not covering upper bound users. In fact, producers and regulators shall guarantee the safety of both average and upper bound users when designing their products. The present model represents a powerful green and sustainable chemistry tool applicable by industries to assess whether the chemicals present in their products could pose children at risk as well as to evaluate potential safer alternatives during the design process.

## Supplementary information

Supplementary tables and figures

Supporting Information

Supplementary Material
