# Supplementary material for: Estimating mouthing exposure to chemicals in children’s products
Source: J Expo Sci Environ Epidemiol. 2021 Jun 29;32(1):94–102. doi: 10.1038/s41370-021-00354-0 (PMC8770116; doi:10.1038/s41370-021-00354-0)
Supplement: Supplementary file 1 — Supplementary tables and figures [file 41370_2021_354_MOESM1_ESM.pdf]

S-1. Sources used for the chemical migration rates into saliva dataset and calculation of migration rates from experimental settings and results

Table S1. Overview of the considered experimental studies reporting chemical migration rates from toys to saliva.

Study	Products tested	Materials tested	Chemicals tested	
			n	Main chemical classes
Bouma et al. [1]	Soft toys (n = 62)	PVC	6	Phthalates
Simoneau et al. [2]	Standard disks (n = 30)	PVC	5	Phthalates
Babich et al. [3]	Toys and child care articles (n = 63)	PVC, ABS, PE, PP, PS	4	Phthalates
Makkliang et al. [4]	Baby teether (n = 1)	Plastic	3	Phthalates
Chen [5]	Toys (n = 41)	ABS, BS, PEVA, PE, PP, PS, PVC	2	Phthalates
Babich et al. [6]	Children's products	PVC	1	Phthalates
Bremmer et al. [7]	Unspecified object (n = 3)	PVC	1	Phthalates
Özer et al. [8]	Toys (n = 3)	PVC	1	Phthalates
Niino et al. [9]	Toys (n = 5)	PVC	1	Phthalates
Kirchnawy et al. [10]	Toys (n = 31)	PVC	7	Phthalates and alternatives
Choi et al. [11]	Children's products (n = 5)	Hard and soft plastic, EVA, Synthetic leather, PVC, metal,	12	Phthalates and metals
Chen et al. [12]	Toys (n = 1)	PVC	17	Brominated flame retardants
Ionas et al. [13]	ERM-EC591* (n = 1)<br>Toys (n = 2)	PP, hard plastic	10	Brominated flame retardants
Masuck et al. [14]	Toys (n = 7)	PVC	24	Fragrance allergens
Potouridis et al. [15]	Gel-filled baby teethers (n = 3)	EVA	3	Parabens
Xu et al. [16]	Hard toys (n = 40)<br>Pacifiers (n = 74)<br>Teethers (n = 32)<br>Soft rubber toys (n = 44)	Silicone-containing	15	Methylsiloxanes
Wang et al. [17]	Toys (n = 3)	Wood	6	Preservatives
Noguerol-Cal et al. [18]	Toys (n = 13)	PP	9	Azo dyes, antioxidants

*Certified reference material (CRM); ABS = poly(styrene:acrylonitrile:butadiene); BS= poly(styrene:butadiene); P/EVA = poly(ethylene-co-vinyl acetate); PE = polyethylene; PP = polypropylene; PS = polystyrene; PVC = polyvinyl chloride.

Few of the gathered studies did not report directly chemical migration rates from tested products into saliva, but reported the final chemical concentration in saliva or the chemical concentration in the sample migrated to saliva. In these cases, we calculated the chemical migration rates from the experimental settings and results. In case a study reported the final chemical concentration in saliva C_{saliva} (µg/mL), we estimated the migration rate in µg/10cm2/min by applying the following equation:

$$R_{\mathrm{mgr}} = \frac{C_{saliva} \times V_{saliva}}{\frac{A_{sample}}{10} \times t}$$

Where R_{mgr} is the migration rate (µg/10cm^2/min), C_{saliva} is the final chemical concentration in saliva (µg/mL), V_{saliva} is the volume of saliva in which the sample is immerged (mL), A_{sample} is the surface area of the sample in contact with saliva (cm^2) divided by 10 for scaling R_{mgr} to the typical mouthing area of children (10cm^2), and t is the contact time (min).

One case study [17] reported as main result the fraction migrated reported to the mass of the sample (not the mass of chemical in the sample), $f_{migrated}$ (µg$_{\mathrm{chemical}}$/g$_{\mathrm{sample}}$). We therefore estimated the migration rate in µg/10cm2/min applying the following equation:

$$R_{\mathrm{mgr}} = \frac{f_{migrated} \times m_{sample}}{\frac{A_{sample}}{10} \times t}$$

Where $C_{migrated}$ is the concentration of chemical in sample migrated to saliva (µg/g) and m_{sample} is the mass of the sample (g).

S-2. Model for predicting migration into saliva

Estimation of model input parameters

The two key input parameters D_p and K_{pf} are estimated by the following mathematical relationships as a function of both chemical properties and material type. The following quantitative property-property relationship (QPPR) was used to predict D_p from molecular weight, temperature and the material type [20]:

$$\log_{10} D_p - \frac{\tau - 3486}{T} = 6.39 - 2.49 \cdot \log_{10} MW + b$$

where D_p is the diffusion coefficient (m^2/s), MW is molecular weight (g/mol), T is temperature (K), b and τ are material-specific coefficients presented in Table S2. Since the diffusion coefficient is primarily material type related, measured diffusion for at least a few different chemicals are indispensable for predicting the material coefficient, unless a new QSAR would be developed in the future based on material properties rather than material types.

Table S2. Material-specific coefficients for estimating D_p [20].

Material	b	τ
Calcium silicate	1.167	0
Carpet	-1.227	0
Cement	0.330	0
Ethylene-propylene rubbers	-6.323	1676
Flexible PVC	-8.513	1676
General polystyrene (PS)	2.036	-2391
Glass, Stainless steel	-8.570	0
Gypsum and cellulose ceiling tile	-1.238	1676
Gypsum board	-5.770	1676
High density polyethylene (HDPE)	5.113	-2391
High-impact polystyrene (HIPS)	-7.113	0
Methyl methacrylate (MMA) copolymer-medium or low density	-7.726	0
Methyl methacrylate (MMA) homopolymer	-7.845	0
Natural rubber (NR)	-3.599	1676
Other wooden boards	-6.718	1676
Paper	-8.534	1676
Plywood	-5.609	1676
Polyamide (PA)	-5.396	0
Poly acrylnitrile butadiene styrene (ABS), Ethylene vinyl alcohol (EVOH)	-4.968	0
Polychloroprene (CR)	-6.314	1676
Polyethylene (PE, LDPE, LLDPE)	-1.648	0
Polyethylene naphthalate (PEN)	-1.163	-2391
Polyethylene terephthalate (PET)	0.000	-2391
Polystyrene foam (XPS, EPS)	-8.323	1676
Polyurethane-based materials	-7.346	1676
PP copolymer	4.791	-2391
PP homopolymer	4.528	-2391
Rigid polymers	-11.944	1676
Synthetic rubber	-5.929	1676
Vinyl acetate-based polymers	-0.459	0
Vinyl flooring	-6.775	1676
Limited-data material group	-2.256	0

K_{pf} was also predicted by the following QPPR [21]:

$$\log_{10} K_{pf} = -1.36 + 1.06 \cdot \log_{10} K_{ow} - 0.014 \cdot EtOH_{eq} - 0.0066 \cdot \log_{10} K_{ow} \cdot EtOH_{eq} + 866 \cdot \left(\frac{1}{T} - \frac{1}{298.15}\right) + b$$

where b is again a material-specific coefficient presented in Table S3. For material types that do not match any of those in Table S3, a generic QPPR was used to predict the K_{pf}:

$$\log_{10} K_{pf} = -1.96 + 1.16 \cdot \log_{10} K_{ow} - 0.059 \cdot EtOH_{eq} - 0.0079 \cdot \log_{10} K_{ow} \cdot EtOH_{eq} + 805 \cdot \left(\frac{1}{T} - \frac{1}{298.15}\right)$$

Table S3. Material-specific coefficients for estimating K_{pf} [21].

Material	b
Average HDPE/LDPE	0.140
Carbohydrates	-0.570
Ethylene-propylene copolymer	-0.120
Ethylene-vinyl acetate copolymer (EVA)	0.570
High-density polyethylene (HDPE)	0.030
Ionomer (Surlyn 1652, 1702)	-0.040
Low-density polyethylene (LDPE)	0.000
Linear low-density polyethylene (LLDPE)	-1.280
Nylon 6	-0.930
Polyethylene terephthalate (PET)	-0.910
Poly 2-hydroxyethylmethacrylate	0.380
Polycaprolactone related polymers	0.840
Polydimethylsiloxane (PDMS)	-0.010
Polyoxymethylene (POM)	-0.160
Polystyrene (PS)	0.820
Polypropylene (PP) homopolymer	-0.500
Polyvinyl chloride (PVC)	0.480
Silicone rubber	0.380
Starch, Cellulose	-1.850

Uncertainties on the estimated input parameters D_p and K_{ms}

The QPPR (quantitative property-property relationship) for logD_p has adjusted R^2 = 0.93 and S_e = 1.17 [20], meaning that 95% CI of the predicted logD_p is the predicted value +/- 2.30. The QPPR for log K_{ms} has adjusted R^2 = 0.925 and S_e = 0.75 [21], meaning that 95% of the predicted log K_{ms} is the predicted value +/- 1.48. Thus, uncertainties on estimated input parameters amounts to two orders of magnitude for D_p (factor 100) and 1.5 orders of magnitude for K_{ms} (factor 30), which is important, but restricted compared to the substantial variations of up to 9 orders of magnitude for K_{ms}, and up to 21 orders of magnitude for D_p (5.5 orders of magnitude in our study) across substances and materials. There is also uncertainty when the specific material type is not available in the default list of materials and we needed to select a proxy material type that best represents the material used in the mouthing migration experiments or use the available generic regression for K_{ms}. For example, for EVA toys we selected "Vinyl acetate-based polymers" as the material type for D_p estimation, while we used "Generic" for wooden toy. For the mechanistic model, this input

parameter uncertainty is included in the reported model uncertainty and mostly relevant when analyzing prediction on individual substances. While in the case of the regression-based the uncertainty on input parameters could potentially bias the regression model coefficients. This uncertainty is an intrinsic limitation of the present QPPRs, and future research is needed to improve the QPPRs by introducing quantitative material descriptors and using machine learning or other algorithms.

S-3. Pacifier and Doll characteristics

Table S4. Characteristics of the two children's products considered for the exposure and related risks estimates.

Material	Material density [g/cm^3]	Pacifier		Doll	
		Toy mass [g]	Toy volume [cm^3]	Toy mass [g]	Toy volume [cm^3]
PVC	1.30	26.00	20.00	390.00	300.00
EVA	1.30	26.00	20.00	390.00	300.00
Silicone	1.60	32.00	20.00	480.00	300.00
PP	0.92	18.40	20.00	276.00	300.00
Wood	0.57	11.40	20.00	171.00	300.00

* Mass and volume values for pacifiers and dolls are based on averaged values for 10 online-marketed children's products.

** All the possible children's products and materials combinations are showed for comparison purpose only (e.g., wooden pacifier)

S-4. Dataset with harmonized migration rates, mouthing exposure estimates and risk assessment results

Table S5. Substance identifier, experimental information, reported chemical mass fraction, migration rate, chemical and material properties, and results of the exposure and risk assessment for the 437 data points of chemicals found children's products.

Table S5 is provided as separate XLSX file.

S-5. Mechanistic material-saliva migration model

The results of the mechanistic material-saliva migration model adapted from the food packaging model fit well the experimental results, with $R^2 = 0.85$ and $S_e = 0.79$ evaluated on the 1:1 line for the log-transformed values. While, as expected the direct correlations (best fit) for the log-transformed values is slightly higher with $R^2 = 0.89$ and $S_e = 0.69$ (y-intercept $\beta_0 = -0.376$ and slope coefficient $\beta_1 = 0.846$). The lower than 1 slope indicates that the model tends to slightly overestimate the high mouthing rates, i.e. most of the phthalates as reflected on Fig. 4. In addition, we calculated the standard error for each chemical family individually (Table S6), the weighted average of the standard error of 0.79 corresponding to the overall standard error in term of deviation from the 1:1 line.

Table S6. Sample size and standard errors on the Log between measured and predicted migration rates (deviation from the 1:1 line) for individual chemical groups.

Chemical group for testing (excluded from training)	Number of data point	S_e	MAE
Plasticizers	177	0.759	0.580
Polybrominated diphenyl ethers	91	0.869	0.737
Parabens	7	0.407	0.317
Methylsiloxanes	40	0.492	0.423
Chlorophenols	104	0.745	0.694
Fragrances	9	1.029	0.765
Other	4	2.050	1.625
Weighted average	-	0.786	0.650

* MAE: mean absolute error

S-6. Regression-based model

The following equation presents the multiple linear regression model:

$$\log_{10} R_{mgr} = \beta_0 + \beta_1 \log_{10} C_0 + \beta_2 \log_{10} D_p + \beta_3 \log_{10} K_{ow}$$

Where R_{mgr} is the chemical migration rate (µg/10cm^2/min), β_0 is the y-intercept, D_p is the chemical diffusion coefficients within materials (cm^2/s), C_0 is the initial chemical concentration in the material (µg/g), K_{ow} is the octanol-water partitioning coefficient and β_1, β_2 and β_3 are the slope coefficients for each independent variable.

Table S7. Multiple linear regression statistics ($R^2 = 0.89$, $S_e = 0.68$).*

Variable	Symbol	Coefficient	Standard error	p-value
Intercept	β_0	3.231	0.302	8.4×10^{-24}
C_0 slope coefficient	β_1	0.924	0.016	7×10^{-205}
D_p slope coefficient	β_2	0.726	0.037	2.1×10^{-61}
K_{ow} slope coefficient	β_3	-0.058	0.017	8×10^{-4}

* when adding MW, the MW beta coefficient was not significant (p-value=0.79). When using K_{ms} instead of K_{ow} the model was not as performant ($R^2 = 0.86$, $S_e = 0.71$ and p=0.17 for K_{ms} against 8×10^{-4} for K_{ow}). There is a high correlation between K_{ms} and K_{ow} for the experimental data and material, and the available dataset might not enable to identify the most relevant factors. Migration data on materials with larger differences on material coefficient (see Huang and Jolliet 2019 [21]) would be needed to make a definite selection here.

As first step to investigate possible influences of artifacts of the dataset on the model predictive performance we carried out a 10-fold cross-validation with random sampling from the dataset. The results are presented in Table S8.

Table S8. 10-fold cross validation statistics results.

Resample	RMSE	R^2	MAE
1	0.593	0.926	0.460
2	0.708	0.899	0.538
3	0.648	0.920	0.516

4	0.677	0.898	0.533
5	0.667	0.887	0.532
6	0.713	0.881	0.539
7	0.719	0.884	0.542
8	0.619	0.914	0.511
9	0.580	0.928	0.479
10	0.834	0.829	0.689
Average	0.676	0.895	0.534

* RMSE: root-mean-square error; MAE: mean absolute error

Since the 10-fold cross-validation with random sampling from the dataset is not able to fully reflect the actual performances of the model in predicting e.g., additional chemical groups we performed a second cross-validation but with a manual selection of the training and testing samples. The analysis was performed both at chemical group and at data source level, and the results are summarized in Table S9. The results of this analysis show that especially when excluding entire chemical groups (e.g., Polybrominated diphenyl ethers) the performances of the model are substantially influenced. These results support the recommendation of implementing the adapted food packaging model for predicting chemical migration rate for new chemical-material combinations since the adapted food packaging model is fully predictive without any parameter adjustment and shows good predictive powers for a large range of chemical-material combinations.

Table S9. Cross-validation statistics results with manual sampling of chemical groups and data sources. The information in "Resample" column indicates either the chemical group or studies excluded from the training of the dataset and used for testing. The columns "Training" and "Testing" indicate the size of the samples.

Resample	Training	Testing	RMSE	MAE
Chemical group				
Plasticizers	255	177	1.245	1.175
Polybrominated diphenyl ethers	341	91	1.395	1.324
Parabens	425	7	0.678	0.622
Methylsiloxanes	392	40	0.382	0.319
Chlorophenols	328	104	0.679	0.570
Fragrances	423	9	1.107	0.934
Other	428	4	1.299	1.184

Weighted average	-	-	1.041	1.029
Experimental data source				
Babich et al. [6]	408	24	0.558	0.499
Chen [5]	429	3	0.146	0.136
Bouma et al. [1]	386	46	0.246	0.202
Simoneau et al. [2]	413	19	0.301	0.251
Babich et al. [3]	382	50	0.492	0.408
Kirchnawy et al. [10]	401	31	0.825	0.715
Özer et al. [8]	430	2	0.779	0.769
Choi et al. [11]	430	2	1.744	1.591
Chen et al. [12]	416	16	2.020	1.887
Ionas et al. [13]	357	75	1.673	1.651
Potouridis et al. [15]	425	7	0.678	0.622
Xu et al. [16]	392	40	0.382	0.319
Wang et al. [17]	328	104	0.679	0.570
Masuck et al. [14]	423	9	1.107	0.934
Noguerol-Cal et al. [18]	428	4	1.299	1.184
Weighted average	-	-	0.965	0.906
Weighted average plasticizers	-	-	0.545	0.472

* RMSE: root-mean-square error; MAE: mean absolute error

S-7. Analysis of chemical-material properties of influence

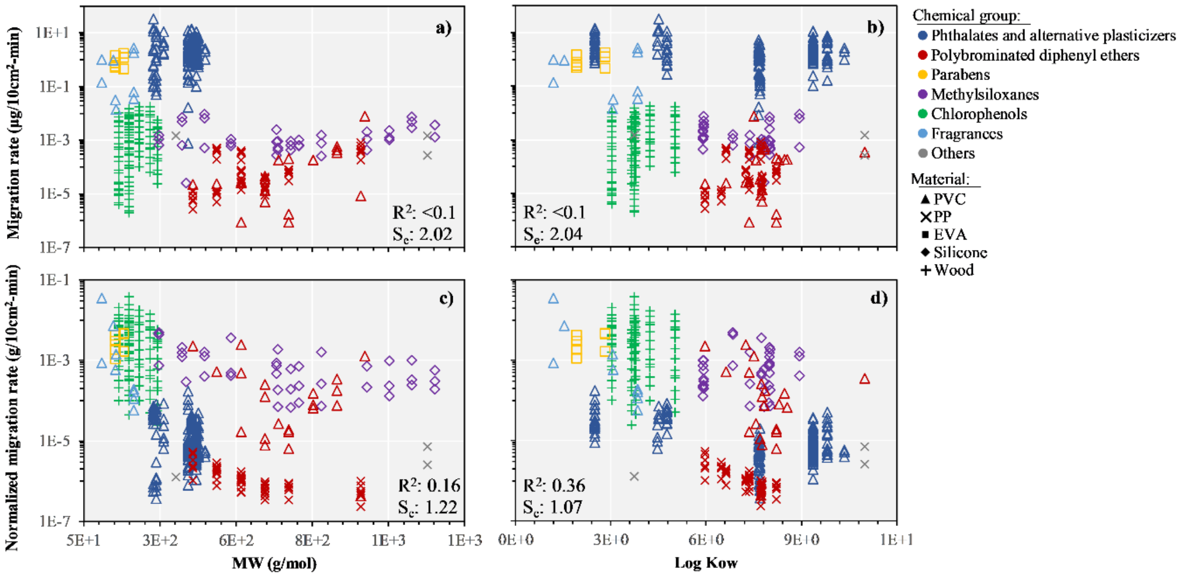

Fig. S1 Measured migration rates as function of Molecular weight (MW) (**a**), and octanol-water partitioning coefficient (K_{ow}) (**b**) and comparison of the ratio between chemical mass fraction and migration rates (normalized migration rates) as a function of MW (**c**) and K_{ow} (**d**). EVA = poly(ethylene-co-vinyl acetate); PP = polypropylene; PVC = polyvinyl chloride.

S-8. Comparison between the predicted and experimental slope

Fig. S2 compares the predicted normalized migration rates (i.e., ratio between migration rate and initial chemical concentration in the product) against the experimental ones. It results in a predictive $R^2 = 0.62$ and a standard error on the Log of $S_e = 0.79$ (no parameter adjustment).

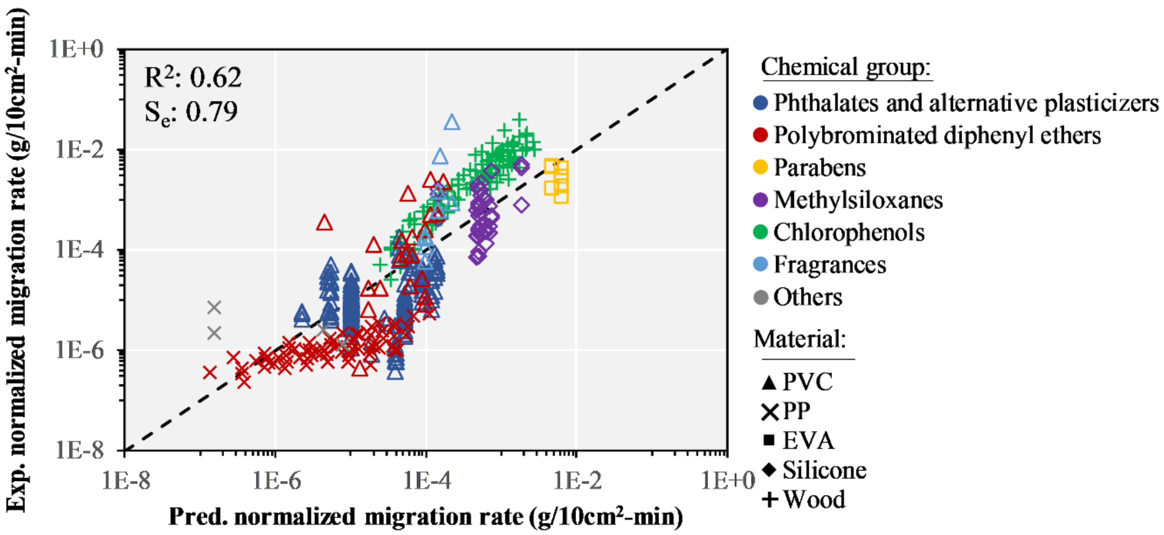

Fig. S2 Comparison between the predicted and experimental slope (migration rate over chemical concentration), differentiating between both chemical groups and material (n=437). The dashed black line represents the 1:1 line. Standard error (S_e) and coefficient of determination (R^2) are evaluated on the log-scale and on the 1:1 line. EVA = poly(ethylene-co-vinyl acetate); PP = polypropylene; PVC = polyvinyl chloride.

S-9. Comparison between mechanistic material-saliva migration model and regression-based model results

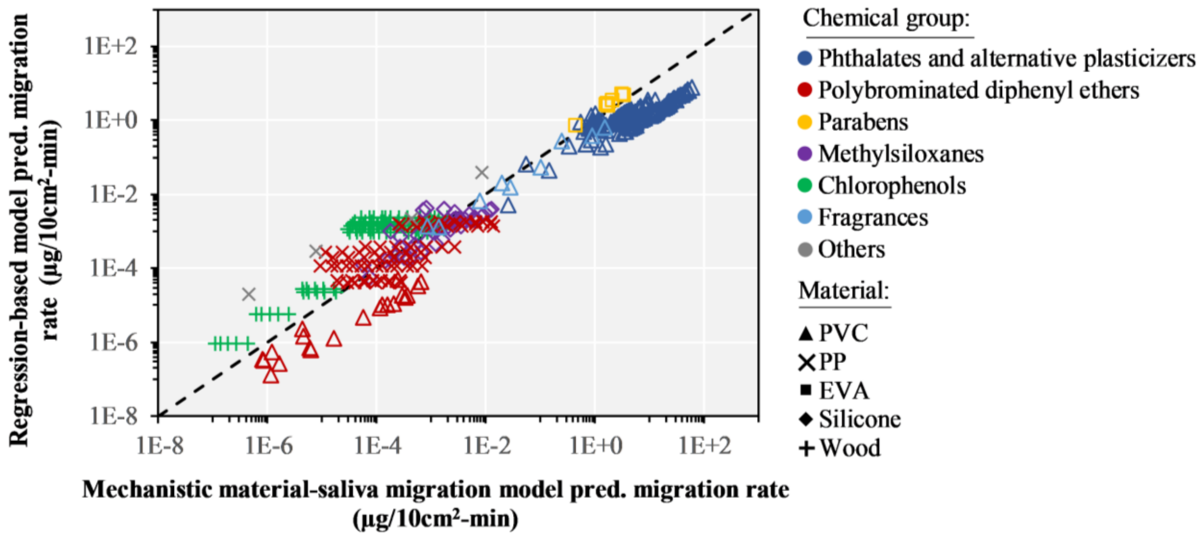

Fig. S3 Comparison between the predicted migration rates with the regression-based model (y-axis) and the predicted migration rates with the mechanistic material-saliva migration model (x-axis), differentiating between both chemical groups and material (n=437). The dashed black line represents the 1:1 line. EVA = poly(ethylene-co-vinyl acetate); PP = polypropylene; PVC = polyvinyl chloride.

S-10. Exposure and risk assessment results

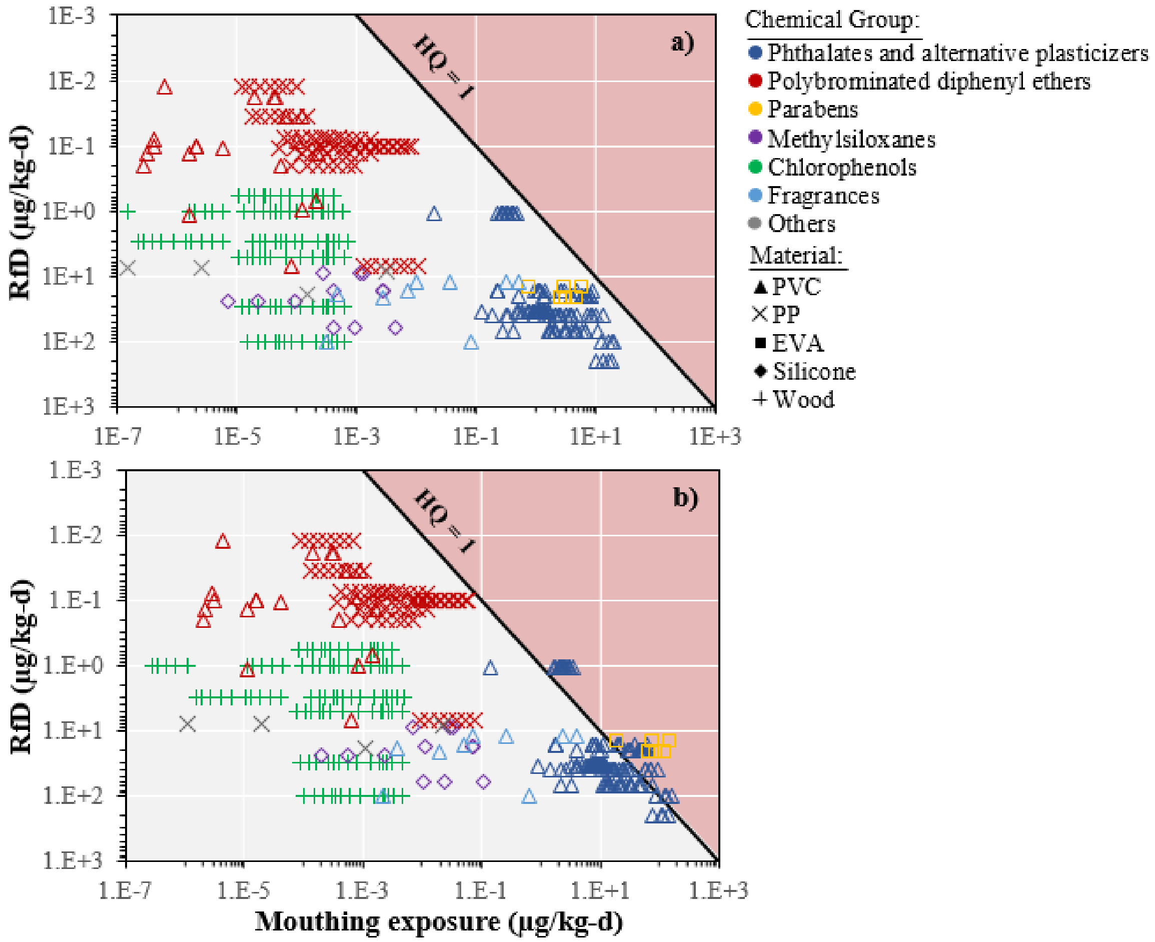

Fig. S4 Non–cancer Reference Doses (inverted axis – high to low RfDs) as a function of mouthing exposures, for the average (**a**) and upper bound (**b**) mouthing exposure duration scenario for 2 to <3 years old children. The black line represent the threshold for hazard quotient of concern (HQ=1). EVA = poly(ethylene-co-vinyl acetate); PP = polypropylene; PVC = polyvinyl chloride.

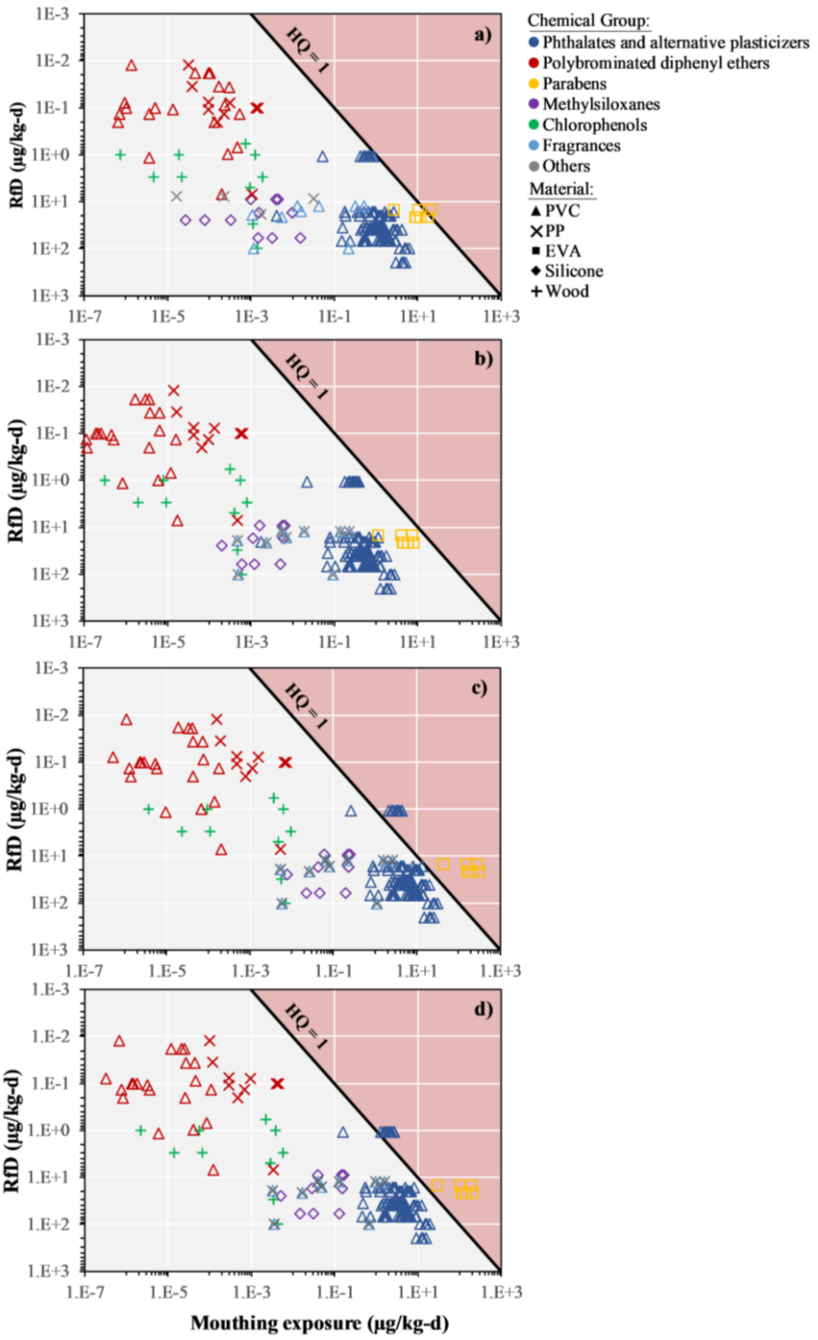

Fig. S5 Risk assessment results for the average mouthing exposure scenario for 3 to <6 months (**a**), for 2 to <3 months old children (**b**), and for the upper bound mouthing behavior scenario for 3 to <6 months (**c**) and for 2 to <3 months old children (**d**). Chemical migration rates estimated with multiple linear regression model. The black line represent the threshold for hazard quotient (HQ) >1. Note that the y-axis is inverted.

S-11. Microsoft Excel workbook

The mechanistic material-saliva migration and the regression-based models are provided as separate Microsoft Excel workbook.

REFERENCES

1. Bouma K, Schakel DJ (2002) Migration of phthalates from PVC toys into saliva simulant by dynamic extraction. Food Addit Contam 19:602–610
2. Simoneau C, Hannaert P, Sarifiannis D (2009) Effect of the nature and concentration of phthalates on their migration from PVC materials under dynamic simulated conditions of mouthing. Jt. Res. Centre, Inst. Heal. Consum. Prot. Eur. Comm. JRC 51604.
3. Babich MA, Bevington C, Dreyfus MA (2020) Plasticizer migration from children's toys, child care articles, art materials, and school supplies. Regul Toxicol Pharmacol 111:104574
4. Makkliang F, Kanatharana P, Thavarungkul P, Thammakhet-Buranachai C (2017) A polypyrrole-chitosan cryogel stir-bead micro-solid phase extractor for the determination of phthalate esters in contact lenses storage solutions and in artificial saliva in contact with baby teethers. Anal Chim Acta 985:69–78
5. Chen S-B Screening of toys for PVC and phthalates migration. June 20, 2002. In: Response to Petition HP 99-1. Request to Ban PVC in Toys and Other Products Intended for Children Five Years of Age and under.
6. Babich MA, Chen SB, Greene MA, Kiss CT, Porter WK, Smith TP, Wind ML, Zamula WW (2004) Risk assessment of oral exposure to diisononyl phthalate from children's products. Regul Toxicol Pharmacol 40:151–167
7. Bremmer, H. J., & Van Veen MP (2002) Children's toys fact sheet.
8. Tümay Özer E, Güer E (2012) Determination of di(2-ethylhexyl) phthalate migration from toys into artificial sweat by gas chromatography mass spectrometry after activated carbon enrichment. Polym Test 31:474–480
9. Niino T, Ishibashi T, Itoh T, Sakai S, Ishiwata H, Yamada T, Onodera S (2002) Comparison of diisononyl phthalate migration from polyvinyl chloride products into human saliva in vivo and into saliva simulant in vitro. J Heal Sci 48:277–281
10. Kirchnawy C, Hager F, Piniella VO, Jeschko M, Washüttl M, Mertl J, Mathieu-Huart A, Rousselle C (2020) Potential endocrine disrupting properties of toys for babies and infants. PLoS One 15:e0231171
11. Choi IS, Choi SC (2014) Contents and Migration of Heavy Metals and Phthalates in Children's Products and Phthalates in Children's Products. J Korean Soc Environ Eng 36:127–138
12. Chen SJ, Ma YJ, Wang J, Chen D, Luo XJ, Mai BX (2009) Brominated flame retardants in children's toys: Concentration, composition, and children's exposure and risk assessment. Environ Sci Technol 43:4200–4206
13. Ionas AC, Ulevicus J, Gómez AB, Brandsma SH, Leonards PEG, van de Bor M, Covaci A (2016) Children's exposure to polybrominated diphenyl ethers (PBDEs) through mouthing toys. Environ Int 87:101–107
14. Masuck I, Hutzler C, Luch A (2011) Estimation of dermal and oral exposure of children to scented toys: Analysis of the migration of fragrance allergens by dynamic headspace GC-MS. J Sep Sci 34:2686–2696
15. Potouridis T, Knauz A, Berger E, Püttmann W (2019) Examination of paraben release from baby teethers through migration tests and GC–MS analysis using a stable isotope dilution assay. BMC Chem 13:70
16. Xu L, Zhi L, Cai Y (2017) Methylsiloxanes in children silicone-containing products from China: Profiles, leaching, and children exposure. Environ Int 101:165–172
17. Wang Z, Ma Q, Bai H, Zhang Q, Cai Y, Lv Q (2019) Migration regularity of six preservatives from wooden children's products to saliva and sweat based on

microstructure-related migration models. Ecotoxicol Environ Saf 173:149–155

18. Noguerol-Cal R, López-Vilariño JM, González-Rodríguez M V., Barral-Losada L (2011) Effect of several variables in the polymer toys additive migration to saliva. Talanta 85:2080–2088
19. Ernstoff AS, Fantke P, Huang L, Jolliet O (2017) High-throughput migration modelling for estimating exposure to chemicals in food packaging in screening and prioritization tools. Food Chem Toxicol 109:428–438
20. Huang L, Fantke P, Ernstoff A, Jolliet O (2017) A quantitative property-property relationship for the internal diffusion coefficients of organic compounds in solid materials. Indoor Air 27:1128–1140
21. Huang L, Jolliet O (2019) A combined quantitative property-property relationship (QPPR) for estimating packaging-food and solid material-water partition coefficients of organic compounds. Sci Total Environ 658:493–500
